# CAR‐NK, a Splendid Strategy for Cancer, Especially for Gynecologic Tumor

**DOI:** 10.1002/iid3.70210

**Published:** 2025-06-17

**Authors:** Yisen Cao, Liying Wang, Liang Wang

**Affiliations:** ^1^ Fujian Medical University; ^2^ Laboratory of Gynecologic Oncology, Fujian Maternity and Child Health Hospital, College of Clinical Medicine for Obstetrics & Gynecology and Pediatrics Fujian Medical University Fuzhou Fujian China; ^3^ Fujian Key Laboratory of Women and Children's Critical Diseases Research, Fujian Maternity and Child Health Hospital (Fujian Women and Children's Hospital) Fuzhou Fujian China; ^4^ Fujian Clinical Research Center for Gynecological Oncology, Fujian Maternity and Child Health Hospital (Fujian Obstetrics and Gynecology Hospital) Fuzhou Fujian China

**Keywords:** CAR‐NK cell, cell therapy, chimeric antigen receptor, gynecological cancers, solid tumor

## Abstract

**Background:**

NK cells are a class of innate lymphocytes capable of nonspecifically killing tumor cells without MHC restriction or prior sensitization. Recent advancements in biotechnology, particularly the development of chimeric antigen receptors (CAR) and related technologies, have enabled targeted tumor cell elimination. CAR endows NK cells with enhanced functionality, with the extracellular domains typically consisting of single‐chain variable fragments (scFv) for targeting specific antigens. CAR‐NK cells have shown excellent results in several preclinical studies and clinical trials for hematologic malignancies. However, their clinical application in the treatment of solid tumors is still insufficient. Current treatments for gynecological cancers primarily involve surgery, chemotherapy, and radiotherapy, all of which often present substantial side effects and variable efficacy. While CAR‐T cell therapy has shown effectiveness in certain gynecological tumors, its clinical application is hindered by severe side effects, such as Cytokine Release Syndrome (CRS) and Graft‐Versus‐Host Disease (GVHD). CAR‐NK cell therapy offers improved safety profiles in clinical applications.

**Objective:**

This review aims to systematically evaluate recent methodological innovations in CAR‐NK engineering and their translational potential in tumor‐targeted treatment, providing valuable insights for clinical trials and studies.

**Methods:**

Electronic databases, including PubMed and Web of Science were searched for relevant literature. Keywords are as follows: CAR‐NK cell; Chimeric antigen receptor; Solid tumor; cell therapy; gynecological cancers.

**Results:**

CAR‐NK engineering has innovations such as multi‐targeted CAR design, gene editing for enhanced persistence, and “off‐the‐shelf” CAR‐NK cells compared to CAR‐T cells.

**Conclusion:**

CAR‐NK cell therapy combines safety and anti‐tumor efficacy, particularly for gynecological cancers.

## 1 Introduction

Natural killer (NK) cells, constituting 10%–15% of peripheral blood lymphocytes and defined by the surface markers CD3 − CD56 + , mediate innate immunity through their ability to perform effective immune surveillance and eliminate cancer cells without antigen‐specific MHC restriction or prior sensitization. Approximately 90% of circulating NK cells are mature CD56dimCD16bright cells, primarily responsible for mediating cytotoxic immune responses. The remaining 10% are immature CD56brightCD16dim NK cells, primarily as cytokine producers (particularly IFN‐γ) mediating immunomodulatory functions [1]. NK cell activation is not a binary process regulated by a single molecular switch or signaling pathway. The equilibrium between activating and inhibitory receptor signaling determines NK cells' cytotoxic potential.aluated in clinical trials for cervical. The tumor microenvironment contains multiple inhibitory mechanisms that impair NK cell function, including immunosuppressive cellular components (dendritic cells, regulatory macrophages, Treg cells, myeloid‐derived suppressor cells (MDSCs), and cancer‐associated fibroblasts); and soluble factors such as indoleamine 2,3‐dioxygenase (IDO), transforming growth factor‐β (TGF‐β), and prostaglandin E2 (PGE2) [2, 3, 4].

CAR‐NK cells are generated by introducing a chimeric antigen receptor (CAR) gene into NK cells. The CAR structure comprises an extracellular recognition domain and an intracellular signaling region. The extracellular domain typically consists of a single‐chain variable fragment (scFv), enabling CAR‐NK cells to specifically recognize target cells. The intracellular region primarily contains multiple signaling domains. The first‐generation CAR molecules relied on a single signaling domain [5], but their limited functionality and poor persistence [6] prompted the development of second‐ and third‐generation CARs. These advanced generations incorporate one or more costimulatory domains, enhancing both the viability and intracellular effector signaling of CAR‐NK cells [7, 8]. Recently, fourth‐generation CAR molecules, which incorporate cytokine payloads, have been developed [9, 10], potentially paving the way for more effective clinical applications.

While early‐stage gynecological cancers are mainly managed through surgery, treatment options for advanced gynecological cancers—whether surgical, radiotherapeutic, chemotherapeutic, targeted, or immunotherapeutic—remain limited [11, 12, 13]. Given the promising therapeutic outcomes of CAR‐based therapies in hematological malignancies, there is strong potential for CAR technology to be adapted for gynecological cancers.

CAR‐T cell therapy has received approval from the U.S. Food and Drug Administration (FDA) for the treatment of hematologic malignancies, demonstrating significant clinical efficacy [14]. However, it is associated with severe side effects, including graft‐versus‐host disease (GVHD) and cytokine release syndrome (CRS) [15, 16, 17]. The release of high levels of inflammatory cytokines, such as IL‐6 and IL‐10, during CAR‐T cell activation can trigger systemic inflammation, enhance immune cell activation, and increase vascular permeability, leading to symptoms like fever and organ damage. Given the tumor contamination in the blood of patients requiring CAR‐T cell therapy, allogeneic CAR‐T cells are often preferred. However, the mismatch between the T‐cell surface receptors and the recipient's MHC molecules can lead to cytotoxicity against the host's cells, initiating GVHD.

In recent years, CAR‐NK cell therapy for solid tumors has garnered increasing attention. While CAR‐NK cells remain underutilized in solid tumor applications, promising preclinical studies and clinical trials suggest their considerable potential. As an alternative to CAR‐T therapy, CAR‐NK cell therapy has shown favorable safety profiles in previous research [18] and comparable efficacy against malignant tumors. This review explores the fundamental role of NK cells, introduces the design and optimization of CAR constructs, and examines both completed and ongoing clinical and preclinical studies on CAR‐NK cells for malignant tumor treatment. A comparative analysis of CAR‐T and CAR‐NK therapies will highlight the potential value of advancing CAR‐NK cell therapies, particularly for the treatment of solid tumors such as gynecologic cancers.

## 2 The Basement Role of NK Cells Against Cancer in the Normal Immune Mechanism

**Figure 1:**
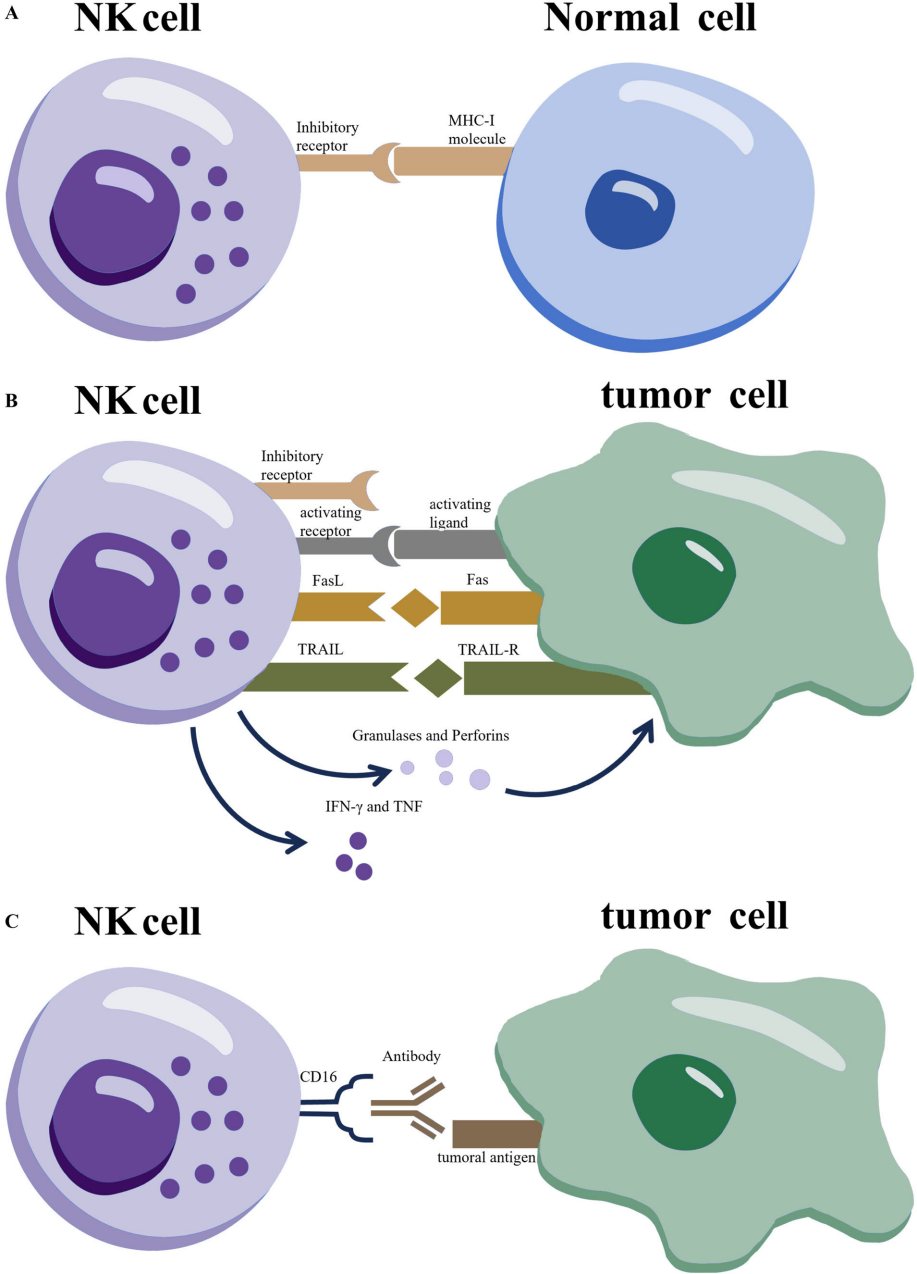
Functions of Natural Killer (NK) Cells | NK cells express a variety of receptors with activating or inhibitory functions, and the balance of activating and inhibitory signaling controls the activation or non‐activation of the cytotoxic effects of NK cells. Normal cells express MHC‐I molecules on their surface, and when inhibitory receptors on NK cells bind to these molecules, the function of NK cells is inhibited and their cytotoxicity is suppressed (A). In abnormal cells (tumor cells, e.g.), their MHC‐I molecules are usually downregulated, while the ligands of NK cell activating receptors (e.g., NKG2DL, etc.) are typically upregulated. The mutual binding of activating ligands and activating receptors (e.g., NKG2D) leads to the absence of inhibitory signals, and the enhancement of activating signals induces the activation of NK cells and cytotoxicity against target cells. The release of cytokines to the recruitment of other immune cells (e.g., macrophages, etc.) enhances the immune response (B). In addition, NK cells are activated through CD16 recognizable antibodies bound on target cells, allowing them to exert cytotoxicity on target cells through ADCC action (C).

NK cells are innate lymphocytes capable of recognizing and lysing transformed or virally infected cells without prior sensitization. Their activation status is determined by the balance between inhibitory and activating receptors. The functional model of NK cells is illustrated in Figure 1. Under normal conditions, inhibitory killer immunoglobulin (Ig)‐like receptors (KIRs) on NK cells interact with major histocompatibility complex class I (MHC I) molecules on healthy cells, preventing NK cell activation [1]. In contrast, virus‐infected and malignant cells often downregulate MHC I, leading to disengagement of KIRs and the subsequent activation of NK cell effector functions against “non‐self” cells. This mechanism of NK cell recognition contrasts with the T‐cell‐mediated response to target cells. Activated NK cells secrete perforin and granzyme; perforin disrupts the target cell membrane, facilitating granzyme entry to induce apoptosis [19]. Additionally, NK cells can trigger apoptosis by binding “death receptors” on target cells with death ligands such as FasL and tumor necrosis factor‐related apoptosis‐inducing ligand (TRAIL) [19], resulting in direct target cell lysis. Although NK cell cytotoxicity is non‐antigen‐specific, it can be directed against abnormal cells via antibody‐dependent cellular cytotoxicity (ADCC). In this process, antibodies bind to antigens on target cells, exposing the Fc region of IgG, which interacts with Fc receptors on NK cells, specifically CD16 and CD32. Activation of these receptors leads to degranulation and the release of cytotoxic granules, inducing apoptosis in target cells [19]. TNF not only promotes tumor cell apoptosis but also stimulates IFNγ secretion from NK cells, enhancing their cytotoxic activity by augmenting the release of granzyme and perforin. In addition to direct cytotoxicity, NK cells recruit T cells and macrophages to infection sites through IFN and TNF production [19]. However, TGF‐β inhibits NK cell function, suggesting that targeting TGF‐β with agents such as YM101 or M7824 may enhance NK cell activity [20, 21]. NK cells are pivotal effector cells of the innate immune system, capable of lysing tumor cells without the need for tumor‐specific antigen presentation.

## 3 The Construct of CAR‐NK Cells and the Differences Between CAR‐NK and CAR‐T Cells

Cell sources for CAR‐T cell therapy include autologous peripheral blood, allogeneic sources, and umbilical cord blood or induced pluripotent stem cell (iPSC) differentiation sources. However, due to the limited availability and complexity of preparing umbilical cord blood and iPSC‐derived sources, these are not considered in this review. The quality of peripheral blood from patients requiring CAR‐T treatment varies, with potential tumor contamination, making allogeneic T cells the preferred choice. However, T cells from allogeneic sources may present a risk of graft‐versus‐host disease (GVHD) due to the mismatch between the TCR of donor T cells and the recipient's MHC molecules, leading to unwanted cytotoxicity against normal tissue. In contrast, NK cell activation is independent of MHC class I molecules, making allogeneic NK cell transplantation a safer alternative, as it does not induce GVHD or other alloimmune or autoimmune toxicities. Additionally, when autologous NK cells are used for overdose therapy, their interaction with MHC molecules on autologous cells produces inhibitory signals, preventing damage to normal tissue [15]. This characteristic enables the use of “off‐the‐shelf” allogeneic NK cells from healthy donors [16].

While the advent of CAR‐T cells has revolutionized tumor immunotherapy, a major side effect to consider is cytokine release syndrome (CRS), where T cells release high levels of pro‐inflammatory cytokines, such as IL‐6 and IL‐10, which can lead to nontumor tissue damage. Due to differences in NK cell activation and signaling pathways, the risk of CRS is significantly lower in NK cell‐based therapies [22].

T cells exhibit a relatively long lifespan, and in the context of the severe toxicities discussed earlier, this prolonged lifespan complicates the management of off‐target effects. To mitigate these risks, suicide genes may need to be incorporated to regulate excessive immune responses. In contrast, NK cells have a shorter lifespan, which naturally reduces the risk of persistent “on‐target, off‐tumor” effects, even without the use of suicide genes [23]. However, the limited survival period of NK cells necessitates further evaluation and enhancement of the durability and overall efficacy of CAR‐NK cell therapy. Potential strategies for improving this are outlined below.

Regarding their cytotoxic mechanisms, CAR‐T cells rely on the CAR to recognize tumor antigens and activate their killing function through the T‐cell receptor (TCR). However, tumor cells often downregulate MHC I molecules on their surfaces, limiting the effectiveness of the TCR in such cases. In contrast, CAR‐NK cells can bypass this limitation. Even when tumor cells downregulate MHC I‐like molecules, CAR‐NK cells can still exert their cytotoxic effects via the “missing self” pathway, leveraging their intrinsic immunity to eliminate tumor cells.

Furthermore, unlike CAR‐T cells, CAR‐NK cells employ intracellular costimulatory domains that are not solely dependent on the CD3ζ pathway. These domains contain three types of immunoreceptor tyrosine‐based activation motifs (ITAMs). DAP12, a specialized junction molecule associated with NK cell receptors, contains an ITAM that interacts with Syk and ZAP70 kinases, enhancing NK cell activation and cytokine release [24], in contrast to the CD3ζ‐based signaling pathway in T cells. Notably, fusing NKG2D as the extracellular recognition domain of CAR with DAP12 as the intracellular signaling domain enhances CAR‐NK cell function, as DAP12 is an endogenous signal that activates the NKG2D receptor [16]. A comparative summary of CAR‐NK cells and CAR‐T cells is presented in Table 1.

**Table 1:** The differences between CAR‐NK and CAR‐T cells.

Perspective	CAR‐T cells	CAR‐NK cells	
Manufacturing	Time‐consuming, complex, and requires personalization	Potential for “off‐the‐shelf” use, easier to manufacture	
Basic immunological characteristics	Restricted by MHC I	MHC I independency	[23]
GVHD	Cause GVHD	Not cause GVHD	[15, 16]
CRS	High incidence of severe CRS	The incidence and severity of CRS is low	[17]
Lifespan	longer lifespan, usually months to years	Shorter lifespan, usually 2 weeks	[25, 26]
Design of intracellular structural domains	CD3z with or without other costimulatory molecular	CD3z with or without other costimulatory molecular DAP12 is more cytotoxic than CD3z	[19]

## 4 The Current Application of CAR‐NK Cells in Cancer

In recent years, CAR‐NK cell therapy has gained increasing attention as a promising treatment for cancer, with a growing number of clinical trials enrolling patients [18]. This trend facilitates a more comprehensive evaluation of the efficacy and safety of CAR‐NK cell therapy. A partial summary of the application of CAR‐NK cell therapy in cancer treatment is provided in Table 2.

**Table 2:** Incomplete summary of CAR‐NK cell clinical trials.

Row	Gov Identifier	Conditions	Status
1	NCT05574608	CD123‐positive acute myeloid leukemia (AML)	Recruiting
2	NCT06045091	relapsed/refractory multiple myeloma or plasma cell leukemia	Recruiting
3	NCT05673447	CD19‐positive diffuse large B cell lymphoma	Recruiting
4	NCT06696846	relapsed/refractory T‐lymphoma and acute myeloid leukemia; CD70 positive	Recruiting
5	NCT06307054	acute myeloid leukemia; CLL1‐positive	Recruiting
6	NCT03692767	Relapsed and Refractory B Cell Lymphoma; CD22‐positive	Recruiting
7	NCT03383978	HER2‐positive glioblastoma or its variant gliosarcoma	Active, not recruiting
8	NCT06464965	the positive expression of CLDN18.2 ≥ 10%；pancreatic cancer and gastric cancer	Recruiting
9	NCT06652243	Glypican‐3 (GPC3)‐Positive Advanced Hepatocellular Carcinoma	Not yet recruiting
10	NCT05922930	at least 1 + TROP2 expression; Ovarian Cancer	Recruiting
11	NCT05703854	CD70‐positive osteosarcoma or mesothelioma	Recruiting

### 4.1 CAR‐NK Cells in Solid Tumors

Several reviews have addressed the use of CAR‐NK cells for cancer treatment [3, 27, 28, 29]. In the case of solid tumors, the diversity of antigens and the increasing variety of cancer types pose challenges. Anti‐HER2‐engineered NK cells have been developed to target breast cancer [30]. For glioblastoma, anti‐HER2‐NK92 cells are being investigated, with a clinical trial currently underway (NCT03383978) [31, 32]. In gastric cancer, anti‐HER2‐NK92 cells have demonstrated synergy with apatinib in preclinical studies [33]. Additionally, clinical trials are recruiting patients for CAR‐NK cells targeting Claudin 18.2 (CLDN18.2) in gastric and pancreatic cancers (NCT06464965). Anti‐Robo1‐CAR‐NK‐92 cells, combined with brachytherapy, have been shown to inhibit pancreatic carcinoma in mouse models [34]. Under the synergistic effect of radiotherapy, anti‐CPG3‐NK92 cells engineered with CXCR2 displayed potent cytotoxicity against hepatocellular carcinoma (HCC), though the antitumor effect varied with different radiation doses [35]. Anti‐GPC3‐NK cells are set to be tested in volunteers with primary HCC in a clinical trial (NCT06652243). In colorectal cancer, NKG2D‐CAR‐NK cells, administered via local infusion to three advanced patients, resulted in tumor regression targeting NKG2D ligands [16]. Anti‐HER1‐CAR‐NK cells showed strong tumor‐killing activity against head and neck squamous cell carcinoma, but upregulation of CD44v6 expression may necessitate a multi‐target combination therapy approach [36].

### 4.2 CAR‐NK Cells in Hematologic Tumors

In hematologic malignancies, CAR‐NK cell immunotherapy has shown promising results. In multiple myeloma (MM) [37, 38], CS1‐CAR‐NK cells were found to effectively inhibit tumor cell proliferation and significantly improve survival in tumor‐bearing mice in an allogeneic transplant model [37]. Additionally, three types of CAR‐NK cells targeting CD19 have demonstrated selective cytotoxicity against tumor cells [38]. GPRC5D and BCMA (NCT06045091) are also being explored as alternative targets [39, 40]. A recent study by Yang et al. [41] revealed that CAR‐NK cells targeting CD19/CD20 exhibited enhanced cytotoxicity against acute lymphoblastic leukemia (ALL), even in the absence of target antigens.

While data from preclinical and clinical trials indicate that CAR‐NK cells show significant efficacy and safety in treating solid tumors [18], several challenges remain to be addressed, which are discussed below.

## 5 The Current Strategies to Improve and Optimize CAR‐NK Cells

**Figure 2:**
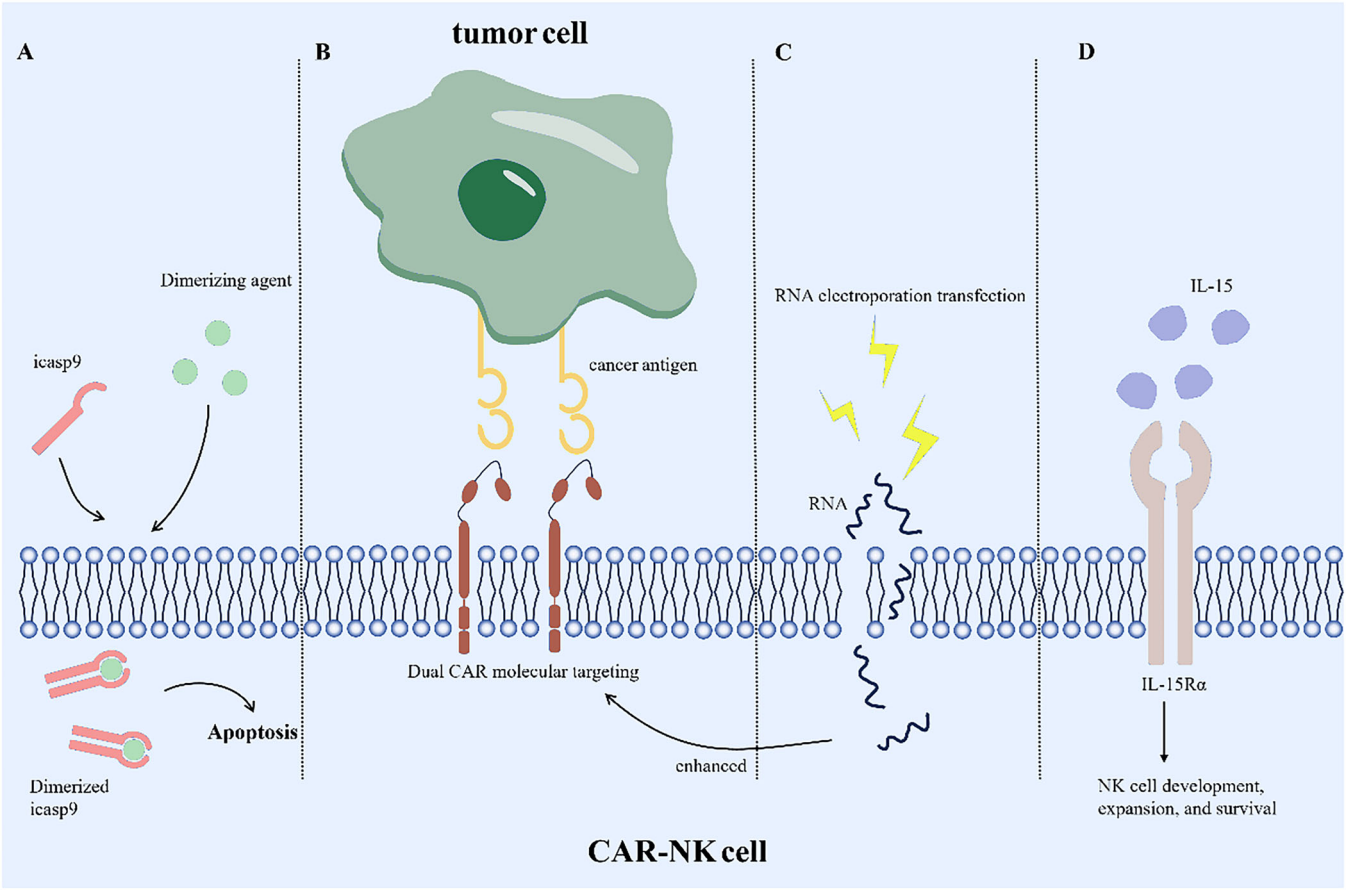
Existing Partial Enhancement Strategies for CAR‐NK Cells | Introduction of the icasp9 suicide gene. When needed, injection of dimerized agents to terminate CAR‐NK cell action (A); dual‐target targeting (B); RNA electroporation transfection to enhance CAR molecular activity (C); IL‐15/IL‐15Rα support to enhance CAR‐NK cell persistence (D).

CAR‐NK cells offer the advantage of avoiding the common issues associated with CAR‐T cells, such as GVHD and CRS. However, enhancing the targeted killing of tumor cells by CAR‐NK cells remains a significant research challenge. This review discusses current strategies aimed at improving CAR‐NK cell efficacy, providing potential directions for future technological advancements. These methods are summarized in Figure 2.

### 5.1 Enhancing Sustainability of CAR‐NK Cells

The relatively short lifespan of CAR‐NK cells, typically around 2 weeks, presents a challenge for their ability to fully accomplish tumor cell elimination. Extending the persistence of CAR‐NK cells in vivo is essential for enhancing their effectiveness in targeting tumor tissue. A recent study on fourth‐generation CAR molecules addressed this by constructing NK cells expressing CAR.19‐IL‐15/IL‐15Rα, which demonstrated enhanced proliferation and strong cytotoxicity against the CD19‐positive Raji cell line [42]. However, further evaluation is necessary to balance prolonged NK cell persistence with the potential risks of nontumor tissue toxicity, as discussed in detail later.

### 5.2 Addressing Low Cancer Antigen Expression in CAR‐NK Cell Therapy

In the absence of overt pathological conditions, tumor cells may shed their specific targeting molecules due to the action of metal shearing enzymes on their surface. This process not only reduces the presence of specific targeting proteins on the tumor cell surface but also results in the masking of activated receptors on the CAR‐NK cell surface. Consequently, the ability of CAR‐NK cells to effectively kill tumor cells is compromised, leading to reduced therapeutic efficacy [43, 44].

To overcome these challenges, one approach involves multitargeting tumor cells to enhance their elimination. A recent study by Zhang et al. developed CAR‐NK cells targeting both NKG2DL and ErbB2 to address the issue of soluble NKG2DL interference [45]. The bispecific CAR structure offers a solution to the problem of downregulated cancer antigens and insufficient precision [46]. Additionally, RNA electroporation transfection has shown promise in addressing potential immune escape pathways by enabling transient CAR expression. This method has been found to temporarily boost CAR activity, enhancing the cytotoxic capabilities of CAR‐NK cells. NKG2D‐CAR‐NK cells targeting NKG2D ligands have been applied to patients with colorectal cancer [16], with electroporation increasing NKG2D expression and improving tumor cell killing. Furthermore, recent reports suggest that lipid nanoparticle (LNP) transfection of umbilical cord blood‐derived NK cells may offer advantages over electroporation, though further evaluation of the receptor function of mRNA‐LNP‐treated NK cells is required [47].

### 5.3 Combating Off‐Target Effects

The strategy of enhancing CAR‐NK cells to overexpress specific ligands in response to low antigen expression in tumor cells introduces a significant risk of off‐target effects. The expression of tumor antigens, albeit in small amounts, in nontumor tissues is an inherent characteristic. Specifically, many target proteins that are highly expressed on the surface of tumor cells are also found in normal tissue cells. For instance, macrophages and dendritic cells also express NKG2DL [48]. Both CAR‐T and CAR‐NK cell therapies are susceptible to off‐target effects, highlighting a potential risk for CAR‐NK cell treatment [49]. While CAR‐NK cells typically have a short lifespan, around 2 weeks [25], limiting potential damage, the possibility of nontumor toxicity remains a concern. Technological advancements, such as making CAR‐NK cells tunable, offer a means to mitigate off‐target effects [50]. Recent research has demonstrated the feasibility of incorporating a suicide gene, such as the icasp9 suicide switch, within the CAR‐NK construct [51]. For patients requiring repeated CAR‐NK cell infusions, nontumor toxicity can be managed by administering chemicals that promptly terminate CAR‐NK cell activity after they have fulfilled their therapeutic role, thus minimizing their impact on healthy tissues [52]. Furthermore, CAR‐NK cells supported by IL‐15 to extend their lifespan can be engineered with the icasp9 suicide gene, offering a refined approach to balance effective tumor cell clearance while minimizing nontumor toxicity.

## 6 The Elusory State of CAR‐NK Cells Against Gynecological Cancers

Cervical, endometrial, and ovarian cancers constitute the most gynecologic malignancy with both high morbidity and mortality. Cervical cancer is the most common gynecologic cancer and the second leading cause of cancer death in women behind breast cancer and the mortality of cervical cancer patients in poor counties is twice that of women in affluent counties [53]. Endometrial cancer is the fourth most common cancer and the fifth most common cause of cancer death in women [54]. Ovarian cancer accounts for 3.6% of all cancer cases but 4.3% of all cancer deaths, which is the second most common cause of gynecologic cancer death next to cervical cancer, the seventh most common and the eighth most common cancer death in women [55]. Since the immunotherapy strategy represented by CAR has shown significant progress in hematologic malignancies and other solid tumors, it is more urgent to conceive the application of immunotherapy in gynecological cancers for women's health.

It is very worth considering where we are and where we are going in the realm of CAR therapy strategies against gynecological cancers. Totally, CAR‐T is more widely studied for gynecological cancers than CAR‐NK cells.

### 6.1 Prospects for the Development of CAR‐NK Cell Therapy in Endometrial Cancer

Endometrial cancer is the leading malignancy of the female reproductive system in developed countries and ranks second in China. The standard first‐line chemotherapy for recurrent or metastatic endometrial cancer is carboplatin combined with paclitaxel; however, patient outcomes remain poor, with a median progression‐free survival of less than 2 years [56]. Despite improvements in survival across a variety of solid tumors, the prognosis for endometrial cancer has not seen significant progress in recent years [57, 58]. With increasing insights into the unique molecular and genomic features of type II endometrial cancers and the evolving understanding of the immune microenvironment in these tumors, there is a pressing need for molecularly targeted therapies or immunotherapies [59]. While the development of biomarker‐driven targeted therapies for endometrial cancer has been slow [60], the advancement of immunotherapies appears even more critical. MISIIR is a promising target due to its specificity for the female reproductive system and could serve as an effective target for endometrial cancer. Recent studies have shown that MISIIR is overexpressed in both ovarian and endometrial cancers but not in normal tissue cells, and CAR‐T cells targeting MISIIR have demonstrated significant success in lysing MISIIR‐overexpressing cervical cancer model mice and patient‐derived tumor cells [61].

### 6.2 Current Status and Development Prospects of CAR‐NK Cell Therapy for Ovarian Cancer

Ovarian cancer, particularly epithelial ovarian cancer, is challenging to diagnose at an early stage, leading to most cases being diagnosed at an advanced stage. As a result, prognosis is often poor due to limited treatment options. Tumor reduction surgery and platinum‐based chemotherapy remain the mainstay treatments; however, recurrence after treatment is common [62], highlighting the urgent need for novel and more effective therapeutic approaches.

The antigen and CAR design strategies used to engineer T cells or NK cells for ovarian cancer, particularly epithelial ovarian cancer, are highly versatile [63, 64, 65, 66, 67, 68]. Progress in antigen‐targeted therapies for ovarian cancer has accelerated due to the extensive identification of specific or associated antigens. For instance, one study developed anti‐CD133‐CD28‐41BB‐CD3ζ‐CAR‐NK92 cells targeting CD133‐positive ovarian cancer cells, while another designed anti‐CD24‐CD28‐41BB‐CD3ζ‐CAR‐NK92 cells to selectively eliminate CD24‐positive ovarian cancer cells. Both CD133 and CD24 are cancer stem cell markers applicable to other cancers as well [64, 67]. Folate receptor alpha (FRα), overexpressed in 90% of ovarian cancers, has been targeted by second‐generation CAR‐NK92 cells, which exhibited strong cytotoxicity against FRα‐positive ovarian cancer cells [63]. Additionally, anti‐mesothelin CAR‐NK cells derived from iPSCs have been shown to inhibit cancer growth in an ovarian cancer xenograft model [68].

### 6.3 Prospects for the Development of CAR‐NK Cell Therapy in Cervical Cancer

CAR‐NK cell therapy has not yet been applied to cervical cancer. However, other immunotherapeutic approaches are being explored, with a primary focus on targeted therapies for HPV‐related antigens, as most cervical cancers are driven by a well‐defined high‐risk HPV‐related pathogenesis. Monoclonal antibodies such as C1P5 (anti‐HPV E6) and TVG701Y (anti‐HPV E7) have been studied and shown to inhibit tumor growth in cervical cancer mouse models [69]. In recent years, the effectiveness of immune checkpoint inhibitors in treating cervical cancer has improved, and combinations of immune checkpoint inhibitors with HPV therapeutic vaccines, chemotherapy, or radiotherapy are being considered for clinical use [70, 71, 72]. Pembrolizumab has received approval for treating PD‐L1‐positive cervical cancer [73]. Several antigens, including GD2, PSMA, Muc1, and mesothelin, have been targeted in CAR‐T cell therapies for cervical cancer (ClinicalTrials.gov identifiers: NCT03356795 and NCT01583686). Additionally, HER2‐targeted CAR‐T cells have been evaluated in clinical trials for cervical cancer, and preclinical studies have focused on CD47 [74, 75].

The relatively limited use of CAR‐T cell therapy in cervical cancer, compared to other solid tumors, can be attributed to the scarcity of specific and exclusive cancer‐associated antigens in this type of cancer. Ideal candidates for CAR targeting should be broadly expressed on cancer cells while being absent or minimally present on normal tissues. HER2, with an overexpression rate of 38%–94% in cervical cancer, and mesothelin, with an overexpression rate of approximately 25%, are the primary antigens currently targeted by CAR‐T cells in cervical cancer [74].

## 7 Conclusion

This review examines the critical role of NK cells and compares CAR‐NK cell therapy with CAR‐T cell therapy. In numerous clinical and preclinical studies, CAR‐NK cells have shown tumor‐targeting efficacy comparable to CAR‐T cells. However, CAR‐NK cells offer distinct advantages, such as their ability to avoid many common toxicities, including CRS and GVHD, during treatment. Despite these promising outcomes, several challenges remain in optimizing CAR‐NK cell therapy. To address the issue of cancer antigen downregulation, strategies such as multi‐target targeting or enhancing CAR expression may be considered. To extend the efficacy of CAR‐NK cells, cytokine support could be explored, and to improve their tunability, the introduction of suicide genes may be a viable solution. As various innovative approaches continue to emerge, the application of CAR‐NK cell therapy in gynecological oncology is still in its early stages. However, with the advancement of various biotechnologies, CAR‐NK therapy, as a prominent form of immunotherapy, holds the potential to herald a new era in the treatment of cancer, offering hope for the cure of a large number of patients.

## Author Contributions

**Yisen Cao:** conceptualization, investigation, methodology, software, writing – original draft, validation, visualization, writing – review and editing, formal analysis, project administration, data curation, supervision, resources. **Liying Wang:** investigation, funding acquisition, writing – original draft. **Liang Wang:** conceptualization, investigation, writing – original draft, validation, visualization, writing – review and editing, formal analysis, project administration, supervision, resources.

## Ethics Statement

The authors have nothing to report.

## Consent

The authors have nothing to report.

## Conflicts of Interest

The authors declare no conflicts of interest.

## Data Availability

The authors have nothing to report.

## References

[1] M. A. Caligiuri , “Human Natural Killer Cells,” Blood 112, no. 3 (August 2008): 461–469. 18650461 10.1182/blood-2007-09-077438 PMC2481557

[2] H. Wang and D. J. Mooney , “Biomaterial‐Assisted Targeted Modulation of Immune Cells in Cancer Treatment,” Nature Materials 17, no. 9 (September 2018): 761–772. 30104668 10.1038/s41563-018-0147-9

[3] W. Hu , G. Wang , D. Huang , M. Sui , and Y. Xu , “Cancer Immunotherapy Based on Natural Killer Cells: Current Progress and New Opportunities,” Frontiers in Immunology 10 (2019): 1205. 31214177 10.3389/fimmu.2019.01205 PMC6554437

[4] S. Sivori , R. Meazza , C. Quintarelli , et al., “NK Cell‐Based Immunotherapy for Hematological Malignancies,” Journal of Clinical Medicine 8, no. 10 (October 2019): 1702. 31623224 10.3390/jcm8101702 PMC6832127

[5] G. Gross , T. Waks , and Z. Eshhar , “Expression of Immunoglobulin‐T‐Cell Receptor Chimeric Molecules as Functional Receptors With Antibody‐Type Specificity,” Proceedings of the National Academy of Sciences 86, no. 24 (December 1989): 10024–10028. 10.1073/pnas.86.24.10024 PMC298636 2513569

[6] A. Bashiri Dezfouli , M. Yazdi , A. G. Pockley , et al., “NK Cells Armed With Chimeric Antigen Receptors (CAR): Roadblocks to Successful Development,” Cells 10, no. 12 (December 2021): 3390. 34943898 10.3390/cells10123390 PMC8699535

[7] A. Biederstädt and K. Rezvani , “Engineering the Next Generation of CAR‐NK Immunotherapies,” International Journal of Hematology 114, no. 5 (November 2021): 554–571. 34453686 10.1007/s12185-021-03209-4 PMC8397867

[8] M. Karvouni , M. Vidal‐Manrique , A. Lundqvist , and E. Alici , “Engineered NK Cells Against Cancer and Their Potential Applications Beyond,” Frontiers in Immunology 13 (2022): 825979. 35242135 10.3389/fimmu.2022.825979 PMC8887605

[9] M. Chmielewski and H. Abken , “TRUCKs: The Fourth Generation of CARs,” Expert Opinion on Biological Therapy 15, no. 8 (2015): 1145–1154. 25985798 10.1517/14712598.2015.1046430

[10] D. D. Feng , X. H. Chen , J. J. Guo , K. K. Wang , X. M. Zhang , and J. M. Gao , “[Preliminary Study of the Fourth‐Generation CAR‐T Cells Targeting CS1 in the Treatment of Refractory and Recurrent Multiple Myeloma],” Zhonghua Zhong Liu Za Zhi 43, no. 6 (June 2021): 657–665. 34289557 10.3760/cma.j.cn112152-20200415-00347

[11] A. Lainé , A. M. Gonzalez‐Lopez , U. Hasan , R. Ohkuma , and I. Ray‐Coquard , “Immune Environment and Immunotherapy in Endometrial Carcinoma and Cervical Tumors,” Cancers 15, no. 7 (March 2023): 2042. 37046702 10.3390/cancers15072042 PMC10093320

[12] Q. Wang , H. Peng , X. Qi , M. Wu , and X. Zhao , “Targeted Therapies in Gynecological Cancers: A Comprehensive Review of Clinical Evidence,” Signal Transduction and Targeted Therapy 5 (July 2020): 137. 32728057 10.1038/s41392-020-0199-6 PMC7391668

[13] R. Pokhriyal , R. Hariprasad , L. Kumar , and G. Hariprasad , “Chemotherapy Resistance in Advanced Ovarian Cancer Patients,” Biomarkers in Cancer 11 (July 2019): 1179299X19860815. 10.1177/1179299X19860815 PMC6613062 31308780

[14] A. Barzegari , F. Salemi , A. Kamyab , et al., “The Efficacy and Applicability of Chimeric Antigen Receptor (CAR) T Cell‐Based Regimens for Primary Bone Tumors: A Comprehensive Review of Current Evidence,” Journal of Bone Oncology 48 (October 2024): 100635. 39381633 10.1016/j.jbo.2024.100635 PMC11460493

[15] B. Rambaldi , G. Rizzuto , A. Rambaldi , and M. Introna , “Genetically Modified and Unmodified Cellular Approaches to Enhance Graft Versus Leukemia Effect, Without Increasing Graft Versus Host Disease: The Use of Allogeneic Cytokine‐Induced Killer Cells,” Frontiers in Immunology 15 (October 2024): 1459175. 39512351 10.3389/fimmu.2024.1459175 PMC11540647

[16] L. Xiao , D. Cen , H. Gan , et al., “Adoptive Transfer of NKG2D CAR mRNA‐Engineered Natural Killer Cells in Colorectal Cancer Patients,” Molecular Therapy 27, no. 6 (June 2019): 1114–1125. 30962163 10.1016/j.ymthe.2019.03.011 PMC6554529

[17] Y. Zhang , W. Zhou , J. Yang , J. Yang , and W. Wang , “Chimeric Antigen Receptor Engineered Natural Killer Cells for Cancer Therapy,” Experimental Hematology & Oncology 12, no. 1 (August 2023): 70. 37563648 10.1186/s40164-023-00431-0 PMC10413722

[18] N. Lamers‐Kok , D. Panella , A. M. Georgoudaki , et al., “Natural Killer Cells in Clinical Development as Non‐Engineered, Engineered, and Combination Therapies,” Journal of Hematology & Oncology 15 (November 2022): 164. 36348457 10.1186/s13045-022-01382-5 PMC9644572

[19] G. Zenere , O. A. Olwenyi , S. N. Byrareddy , and S. E. Braun , “Optimizing Intracellular Signaling Domains for CAR NK Cells in HIV Immunotherapy: A Comprehensive Review,” Drug Discovery Today 24, no. 4 (April 2019): 983–991. 30771481 10.1016/j.drudis.2019.02.002 PMC7065919

[20] Y. Lan , D. Zhang , C. Xu , et al., “Enhanced Preclinical Antitumor Activity of M7824, a Bifunctional Fusion Protein Simultaneously Targeting PD‐L1 and TGF‐β,” Science Translational Medicine 10, no. 424 (January 2018): eaan5488. 29343622 10.1126/scitranslmed.aan5488

[21] M. Yi , J. Zhang , A. Li , et al., “The Construction, Expression, and Enhanced Anti‐Tumor Activity of YM101: A Bispecific Antibody Simultaneously Targeting TGF‐β and PD‐L1,” Journal of Hematology & Oncology 14 (February 2021): 27. 33593403 10.1186/s13045-021-01045-x PMC7885589

[22] H. Ebrahimiyan , A. Tamimi , B. Shokoohian , et al., “Novel Insights in CAR‐NK Cells Beyond CAR‐T Cell Technology; Promising Advantages,” International Immunopharmacology 106 (May 2022): 108587. 35149294 10.1016/j.intimp.2022.108587

[23] A. G. Pockley , P. Vaupel , and G. Multhoff , “NK Cell‐Based Therapeutics for Lung Cancer,” Expert Opinion on Biological Therapy 20, no. 1 (November 2019): 23–33, 10.1080/14712598.2020.1688298. 31714156

[24] K. Töpfer , M. Cartellieri , S. Michen , et al., “DAP12‐Based Activating Chimeric Antigen Receptor for NK Cell Tumor Immunotherapy,” Journal of Immunology 194, no. 7 (April 2015): 3201–3212. 10.4049/jimmunol.1400330 25740942

[25] J. S. Miller , Y. Soignier , A. Panoskaltsis‐Mortari , et al., “Successful Adoptive Transfer and In Vivo Expansion of Human Haploidentical NK Cells in Patients With Cancer,” Blood 105, no. 8 (April 2005): 3051–3057. 15632206 10.1182/blood-2004-07-2974

[26] A. Valeri , A. García‐Ortiz , E. Castellano , et al., “Overcoming Tumor Resistance Mechanisms in CAR‐NK Cell Therapy,” Frontiers in Immunology 13 (August 2022): 953849. 35990652 10.3389/fimmu.2022.953849 PMC9381932

[27] S. Kloess , A. Kretschmer , L. Stahl , S. Fricke , and U. Koehl , “CAR‐Expressing Natural Killer Cells for Cancer Retargeting,” Transfusion Medicine and Hemotherapy 46, no. 1 (February 2019): 4–13. 31244577 10.1159/000495771 PMC6558329

[28] L. Wang , M. Dou , Q. Ma , R. Yao , and J. Liu , “Chimeric Antigen Receptor (CAR)‐Modified NK Cells Against Cancer: Opportunities and Challenges,” International Immunopharmacology 74 (September 2019): 105695. 31254958 10.1016/j.intimp.2019.105695

[29] K. Rezvani , R. Rouce , E. Liu , and E. Shpall , “Engineering Natural Killer Cells for Cancer Immunotherapy,” Molecular Therapy 25, no. 8 (August 2017): 1769–1781. 28668320 10.1016/j.ymthe.2017.06.012 PMC5542803

[30] A. Kruschinski , A. Moosmann , I. Poschke , et al., “Engineering Antigen‐Specific Primary Human NK Cells Against HER‐2 Positive Carcinomas,” Proceedings of the National Academy of Sciences 105, no. 45 (November 2008): 17481–17486. 10.1073/pnas.0804788105 PMC2582261 18987320

[31] P. Nowakowska , A. Romanski , N. Miller , et al., “Clinical Grade Manufacturing of Genetically Modified, CAR‐Expressing NK‐92 Cells for the Treatment of ErbB2‐Positive Malignancies,” Cancer Immunology, Immunotherapy 67, no. 1 (January 2018): 25–38. 28879551 10.1007/s00262-017-2055-2 PMC11028154

[32] C. Zhang , M. C. Burger , L. Jennewein , et al., “ErbB2/HER2‐Specific NK Cells for Targeted Therapy of Glioblastoma,” JNCI: Journal of the National Cancer Institute 108, no. 5 (December 6, 2015), 10.1093/jnci/djv375. 26640245

[33] X. Wu and S. Huang , “HER2‐specific Chimeric Antigen Receptor‐Engineered Natural Killer Cells Combined With Apatinib for the Treatment of Gastric Cancer,” Bulletin du Cancer 106, no. 11 (November 2019): 946–958. 31711572 10.1016/j.bulcan.2019.03.012

[34] N. Xia , P. Haopeng , J. Gong , et al., “Robo1‐specific CAR‐NK Immunotherapy Enhances Efficacy of (125)I Seed Brachytherapy in an Orthotopic Mouse Model of Human Pancreatic Carcinoma,” Anticancer Research 39, no. 11 (November 2019): 5919–5925. 31704816 10.21873/anticanres.13796

[35] X. Lin , Z. Liu , X. Dong , et al., “Radiotherapy Enhances the Anti‐Tumor Effect of CAR‐NK Cells for Hepatocellular Carcinoma,” Journal of Translational Medicine 22 (October 2024): 929. 39396988 10.1186/s12967-024-05724-4 PMC11472550

[36] J. Nowak , M. Bentele , I. Kutle , et al., “CAR‐NK Cells Targeting HER1 (EGFR) Show Efficient Anti‐Tumor Activity Against Head and Neck Squamous Cell Carcinoma (HNSCC),” Cancers 15, no. 12 (June 2023): 3169. 37370779 10.3390/cancers15123169 PMC10296665

[37] J. Chu , Y. Deng , D. M. Benson , et al., “CS1‐specific Chimeric Antigen Receptor (CAR)‐Engineered Natural Killer Cells Enhance In Vitro and In Vivo Antitumor Activity Against Human Multiple Myeloma,” Leukemia 28, no. 4 (April 2014): 917–927. 24067492 10.1038/leu.2013.279 PMC3967004

[38] S. Oelsner , M. E. Friede , C. Zhang , et al., “Continuously Expanding CAR NK‐92 Cells Display Selective Cytotoxicity Against B‐Cell Leukemia and Lymphoma,” Cytotherapy 19, no. 2 (February 2017): 235–249. 27887866 10.1016/j.jcyt.2016.10.009

[39] S. F. Cho , L. Lin , L. Xing , et al., “BCMA‐Targeting Therapy: Driving a New Era of Immunotherapy in Multiple Myeloma,” Cancers 12, no. 6 (June 2020): 1473. 32516895 10.3390/cancers12061473 PMC7352710

[40] J. Xia , Z. Li , and K. Xu , “Immunotherapies Targeting GPRC5D in Relapsed or Refractory Multiple Myeloma: Latest Updates From 2022 ASH Annual Meeting,” Journal of Hematology & Oncology 16, no. 1 (June 2023): 60. 37277826 10.1186/s13045-023-01461-1 PMC10240779

[41] N. Yang , C. Zhang , Y. Zhang , et al., “CD19/CD20 Dual‐Targeted Chimeric Antigen Receptor‐Engineered Natural Killer Cells Exhibit Improved Cytotoxicity Against Acute Lymphoblastic Leukemia,” Journal of Translational Medicine 22 (March 2024): 274. 38475814 10.1186/s12967-024-04990-6 PMC10935961

[42] R. N. Silvestre , J. Eitler , J. T. C. de Azevedo , et al., “Engineering NK‐CAR.19 Cells With the IL‐15/IL‐15Rα Complex Improved Proliferation and Anti‐Tumor Effect In Vivo,” Frontiers in Immunology 14 (September 2023): 1226518. 37818365 10.3389/fimmu.2023.1226518 PMC10561086

[43] N. Nausch and A. Cerwenka , “NKG2D Ligands in Tumor Immunity,” Oncogene 27, no. 45 (October 2008): 5944–5958. 18836475 10.1038/onc.2008.272

[44] V. Groh , J. Wu , C. Yee , and T. Spies , “Tumour‐Derived Soluble MIC Ligands Impair Expression of NKG2D and T‐Cell Activation,” Nature 419, no. 6908 (October 2002): 734–738. 12384702 10.1038/nature01112

[45] C. Zhang , J. Röder , A. Scherer , et al., “Bispecific Antibody‐Mediated Redirection of NKG2D‐CAR Natural Killer Cells Facilitates Dual Targeting and Enhances Antitumor Activity,” Journal for Immunotherapy of Cancer 9, no. 10 (October 2021): e002980. 34599028 10.1136/jitc-2021-002980 PMC8488744

[46] R. J. Cronk , J. Zurko , and N. N. Shah , “Bispecific Chimeric Antigen Receptor T Cell Therapy for B Cell Malignancies and Multiple Myeloma,” Cancers 12, no. 9 (September 2020): 2523. 32899464 10.3390/cancers12092523 PMC7564024

[47] S. Douka , L. E. Brandenburg , C. Casadidio , et al., “Lipid Nanoparticle‐Mediated Messenger RNA Delivery for Ex Vivo Engineering of Natural Killer Cells,” Journal of Controlled Release 361 (September 2023): 455–469. 37567506 10.1016/j.jconrel.2023.08.014

[48] A. Stojanovic , M. P. Correia , and A. Cerwenka , “The NKG2D/NKG2DL Axis in the Crosstalk Between Lymphoid and Myeloid Cells in Health and Disease,” Frontiers in Immunology 9 (2018): 827. 29740438 10.3389/fimmu.2018.00827 PMC5924773

[49] T. Shum , R. L. Kruse , and C. M. Rooney , “Strategies for Enhancing Adoptive T‐Cell Immunotherapy Against Solid Tumors Using Engineered Cytokine Signaling and Other Modalities,” Expert Opinion on Biological Therapy 18, no. 6 (June 2018): 653–664. 29727246 10.1080/14712598.2018.1473368 PMC6084433

[50] A. Di Stasi , S. K. Tey , G. Dotti , et al., “Inducible Apoptosis as a Safety Switch for Adoptive Cell Therapy,” New England Journal of Medicine 365, no. 18 (November 2011): 1673–1683. 22047558 10.1056/NEJMoa1106152 PMC3236370

[51] B. Yang , X. Wang , X. Wei , and J. Ma , “Development of a Novel HER2‐CAR Monocyte Cell Therapy With Controllable Proliferation and Enhanced Anti‐Tumor Efficacy,” Chinese Medical Journal 137, no. 21 (November 2024): 2590–2602. 38243698 10.1097/CM9.0000000000002944 PMC11557030

[52] S. Oelsner , A. Waldmann , A. Billmeier , et al., “Genetically Engineered CAR NK Cells Display Selective Cytotoxicity Against FLT3‐positive B‐ALL and Inhibit In Vivo Leukemia Growth,” International Journal of Cancer 145, no. 7 (October 2019): 1935–1945. 30860598 10.1002/ijc.32269

[53] R. L. Siegel , K. D. Miller , and A. Jemal , “Cancer Statistics, 2019,” CA: A Cancer Journal for Clinicians 69, no. 1 (January 2019): 7–34. 30620402 10.3322/caac.21551

[54] R. A. Brooks , G. F. Fleming , R. R. Lastra , et al., “Current Recommendations and Recent Progress in Endometrial Cancer,” CA: A Cancer Journal for Clinicians 69, no. 4 (July 2019): 258–279. 31074865 10.3322/caac.21561

[55] S. Lheureux , M. Braunstein , and A. M. Oza , “Epithelial Ovarian Cancer: Evolution of Management in the Era of Precision Medicine,” CA: A Cancer Journal for Clinicians 69, no. 4 (July 2019): 280–304. 31099893 10.3322/caac.21559

[56] K. H. Lu and R. R. Broaddus , “Endometrial Cancer,” New England Journal of Medicine 383, no. 21 (November 2020): 2053–2064. 33207095 10.1056/NEJMra1514010

[57] J. N. McAlpine , S. M. Temkin , and H. J. Mackay , “Endometrial Cancer: Not Your Grandmother's Cancer,” Cancer 122, no. 18 (September 2016): 2787–2798. 27308732 10.1002/cncr.30094

[58] Y. L. Wan , R. Beverley‐Stevenson , D. Carlisle , et al., “Working Together to Shape the Endometrial Cancer Research Agenda: The Top Ten Unanswered Research Questions,” Gynecologic Oncology 143, no. 2 (November 2016): 287–293. 27593736 10.1016/j.ygyno.2016.08.333

[59] Y. C. Lee , S. Lheureux , and A. M. Oza , “Treatment Strategies for Endometrial Cancer: Current Practice and Perspective,” Current Opinion in Obstetrics & Gynecology 29, no. 1 (February 2017): 47–58. 27941361 10.1097/GCO.0000000000000338

[60] R. C. Arend , B. A. Jones , A. Martinez , and P. Goodfellow , “Endometrial Cancer: Molecular Markers and Management of Advanced Stage Disease,” Gynecologic Oncology 150, no. 3 (September 2018): 569–580. 29843906 10.1016/j.ygyno.2018.05.015

[61] A. Rodriguez‐Garcia , P. Sharma , M. Poussin , et al., “CAR T Cells Targeting MISIIR for the Treatment of Ovarian Cancer and Other Gynecologic Malignancies,” Molecular Therapy 28, no. 2 (February 2020): 548–560. 31870622 10.1016/j.ymthe.2019.11.028 PMC7001088

[62] C. Lliberos , G. Richardson , and A. Papa , “Oncogenic Pathways and Targeted Therapies in Ovarian Cancer,” Biomolecules 14, no. 5 (May 2024): 585. 38785992 10.3390/biom14050585 PMC11118117

[63] X. Ao , Y. Yang , W. Li , et al, “Anti‐αFR CAR‐Engineered NK‐92 Cells Display Potent Cytotoxicity Against αFR‐positive Ovarian Cancer,” Journal of immunotherapy (Hagerstown, Md: 1997) 42, no. 8 (October 2019): 284–296. 31261167 10.1097/CJI.0000000000000286 PMC6735933

[64] R. Klapdor , S. Wang , M. Morgan , et al., “Characterization of a Novel Third‐Generation Anti‐CD24‐CAR Against Ovarian Cancer,” International Journal of Molecular Sciences 20, no. 3 (February 2019): 660. 30717444 10.3390/ijms20030660 PMC6387114

[65] V. Jindal , E. Arora , S. Gupta , A. Lal , M. Masab , and R. Potdar , “Prospects of Chimeric Antigen Receptor T Cell Therapy in Ovarian Cancer,” Medical Oncology 35, no. 5 (April 2018): 70. 29651744 10.1007/s12032-018-1131-6

[66] Y. Pang , X. Hou , C. Yang , Y. Liu , and G. Jiang , “Advances on Chimeric Antigen Receptor‐Modified T‐Cell Therapy for Oncotherapy,” Molecular Cancer 17, no. 1 (May 2018): 91. 29769134 10.1186/s12943-018-0840-y PMC5956614

[67] R. Klapdor , S. Wang , U. Hacker , et al., “Improved Killing of Ovarian Cancer Stem Cells by Combining a Novel Chimeric Antigen Receptor‐Based Immunotherapy and Chemotherapy,” Human Gene Therapy 28, no. 10 (2017): 886–896. 28836469 10.1089/hum.2017.168

[68] Y. Li , D. L. Hermanson , B. S. Moriarity , and D. S. Kaufman , “Human iPSC‐Derived Natural Killer Cells Engineered With Chimeric Antigen Receptors Enhance Anti‐Tumor Activity,” Cell Stem Cell 23, no. 2 (August 2018): 181–192 e5. 30082067 10.1016/j.stem.2018.06.002 PMC6084450

[69] Z. Jiang , J. Albanese , J. Kesterson , et al., “Monoclonal Antibodies Against Human Papillomavirus E6 and E7 Oncoproteins Inhibit Tumor Growth in Experimental Cervical Cancer,” Translational Oncology 12, no. 10 (October 2019): 1289–1295. 31325765 10.1016/j.tranon.2019.06.003 PMC6642219

[70] O. Marinelli , D. Annibali , C. Aguzzi , et al., “The Controversial Role of PD‐1 and Its Ligands in Gynecological Malignancies,” Frontiers in Oncology 9 (2019): 1073. 31681606 10.3389/fonc.2019.01073 PMC6803534

[71] T. Shibata , B. J. Lieblong , T. Sasagawa , and M. Nakagawa , “The Promise of Combining Cancer Vaccine and Checkpoint Blockade for Treating HPV‐Related Cancer,” Cancer Treatment Reviews 78 (August 2019): 8–16. 31302573 10.1016/j.ctrv.2019.07.001 PMC6710123

[72] S. J. Otter , J. Chatterjee , A. J. Stewart , and A. Michael , “The Role of Biomarkers for the Prediction of Response to Checkpoint Immunotherapy and the Rationale for the Use of Checkpoint Immunotherapy in Cervical Cancer,” Clinical Oncology (Royal College of Radiologists) (London) (July 2019). 10.1016/j.clon.2019.07.003 31331818

[73] G. Marret , E. Borcoman , and C. Le Tourneau , “Pembrolizumab for the Treatment of Cervical Cancer,” Expert Opinion on Biological Therapy 19, no. 9 (September 2019): 871–877. 31322926 10.1080/14712598.2019.1646721

[74] A. Rodriguez‐Garcia , N. G. Minutolo , J. M. Robinson , and D. J. Powell , “T‐Cell Target Antigens Across Major Gynecologic Cancers,” Gynecologic Oncology 145, no. 3 (June 2017): 426–435. 28377094 10.1016/j.ygyno.2017.03.510

[75] V. Golubovskaya , R. Berahovich , H. Zhou , et al., “CD47‐CAR‐T Cells Effectively Kill Target Cancer Cells and Block Pancreatic Tumor Growth,” Cancers 9, no. 10 (October 2017): 139. 29065481 10.3390/cancers9100139 PMC5664078

